# Multidrug-Resistant Urinary Tract Infections in Pregnant Patients and Their Association with Adverse Pregnancy Outcomes—A Retrospective Study

**DOI:** 10.3390/jcm13226664

**Published:** 2024-11-06

**Authors:** Gabriel-Ioan Anton, Liliana Gheorghe, Viorel-Dragos Radu, Ioana-Sadiye Scripcariu, Ingrid-Andrada Vasilache, Alexandru Carauleanu, Iustina-Solomon Condriuc, Razvan Socolov, Pavel Onofrei, Andreea-Ioana Pruteanu, Ramona-Gabriela Ursu, Tudor Gisca, Demetra Socolov

**Affiliations:** 1 Department of Mother and Child Care, “Grigore T. Popa” University of Medicine and Pharmacy Iasi, 700115 Iasi, Romania; antongabrielioan@gmail.com (G.-I.A.);; 2 Surgical Department, Faculty of Medicine, “Grigore T. Popa” University of Medicine and Pharmacy, 700115 Iasi, Romania; 3 Urology Department, “Grigore T. Popa” University of Medicine and Pharmacy, 700115 Iasi, Romania; onofrei.pavel@gmail.com; 4 Department of Microbiology, “Grigore T. Popa” University of Medicine and Pharmacy Iasi, 700115 Iasi, Romania

**Keywords:** multidrug-resistant bacteria, urinary tract infection, risk factors, pregnancy outcomes

## Abstract

**Background/Objectives**: Multidrug-resistant urinary tract infections (MDR UTIs) constitute an important public health problem, especially in pregnant patients. The aim of this retrospective study was to characterize the bacterial spectrum and the profile of microbial resistance in cases of UTIs occurring in pregnant women, as well as their impact on obstetrical and neonatal outcomes. **Methods**: A total of 371 pregnant patients with UTIs were included in the analysis and were segregated into the following groups based on the type of bacterial resistance to antibiotics: MDR UTIs (70 patients, group 1), UTIs resistant to one class of antibiotics (108 patients, group 2), UTIs resistant to two classes of antibiotics (102 patients, group 3), and sensitive UTIs (91 patients, group 4). We used descriptive statistics for characterizing and comparing the microbial spectrum and the clinical characteristics of the patients. A multinomial logistic regression model for evaluating the relationship between the type of urinary tract infection and adverse obstetric or neonatal outcomes was employed. **Results**: In the case of MDR UTIs, the bacterial spectrum mainly included *Escherichia coli*, *Enterococcus faecalis*, and *Klebsiella species*. We found almost universal resistance to ampicillin. Our data confirmed an increased risk of preterm birth, premature rupture of membranes, neonatal respiratory distress syndrome, and neonatal intensive care unit admission for patients with MDR infections. **Conclusions**: The increased incidence of pathogens resistant to commonly used antibiotic classes in pregnancy suggests the need for the development of local and national protocols that adapt therapeutic and prophylactic regimens to clinical realities.

## 1. Introduction

Multidrug resistance of uropathogens constitutes an important public health problem, especially for the subgroup of pregnant patients, who, due to their particular physiologic state, are more prone to developing urinary tract infections. The 2022 Global Antimicrobial Resistance and Use Surveillance System (GLASS) report emphasizes increasing resistance trends among common bacterial infections [1]. The median reported rates of 42% for third-generation cephalosporin-resistant *Escherichia coli (E. coli)* and 35% for methicillin-resistant *Staphylococcus aureus* (MRSA) across 76 nations are a significant concern. In 2020, 20% of urinary tract infections attributed to E. coli had diminished sensitivity to conventional antibiotics such as ampicillin, co-trimoxazole, and fluoroquinolones. This complicates the proper treatment of prevalent infections [1]. Moreover, it was estimated that in 2021 approximately 1.14 million deaths were attributable to multidrug-resistant bacterial infections [2].

Centers for Disease Control and Prevention (CDC) and European Centre for Disease Prevention and Control (ECDC) described three types of antimicrobial resistance: MDR (multidrug resistance), described as resistance to at least one agent from three or more antibiotic classes; XDR (extensively drug-resistant), described as resistance to the majority of antibiotic classes, except from some agents included in a maximum of two antibiotic classes; and PDR (pandrug-resistant), characterized by resistance to all agents from all antimicrobial classes [3].

There has not been a standard approach to determining the types, classes, or groups of antimicrobial agents that should be used to define MDR, XDR, and PDR. The expert group of the European Society of Clinical Microbiology and Infectious Diseases has developed ‘antimicrobial categories’ for each organism or group of organisms with the intention of placing antimicrobial agents into more therapeutically relevant groups [4]. These new categories are listed in specific tables along with the proposed relevant antimicrobial agents for testing antimicrobial sensitivity for each organism or group of organisms. Regarding *Staphylococcus aureus* (SA), experts have defined MDR as either an MRSA infection or an SA infection that is resistant to at least one antimicrobial agent from at least three different classes of antibiotics [4].

Understanding the multiple mechanisms of bacterial resistance to antibiotics is crucial for developing effective treatment strategies and addressing the growing threat of antibiotic-resistant infections. This knowledge can inform clinical practices and guide research into new antimicrobial agents and therapeutic approaches. There are several mechanisms that facilitate the occurrence of multidrug resistance in microorganisms. A series of spontaneous mutations in the bacterial genome can lead to structural and/or functional changes in the targets of antibiotics [5]. These mutations may alter the bacterial ribosome, enzymes, or cell wall components, diminishing the effectiveness of antibiotics [5]. For example, mutations in the gyrA gene can lead to fluoroquinolone resistance [6,7].

Bacteria can transfer resistance genes through conjugation, a process in which genetic material (plasmids) containing antibiotic resistance genes is directly passed from one bacterium to another [8]. This horizontal gene transfer allows for the rapid spread of resistance traits among bacterial populations, contributing to the emergence of multidrug-resistant strains [9]. Bacteria can uptake genetic material from their environment, including DNA released by dead bacteria, which may contain antibiotic resistance genes [10].

Bacteriophages can transfer DNA between bacteria, potentially carrying antibiotic resistance genes [11]. This transduction enables the exchange of genetic material not only between closely related species but also among diverse bacterial strains, further driving the evolution of resistance. Bacteria can develop specialized transport proteins that actively pump antibiotics out of the bacterial cell before they can exert their effects [12]. These efflux pumps can provide resistance to a wide variety of antibiotics, including tetracyclines and fluoroquinolones. For instance, the AcrAB-TolC efflux system in E. coli plays a significant role in multidrug resistance [13]. Bacteria within biofilms may exhibit increased resistance to antibiotics due to reduced penetration of the drugs within the biofilm and modified bacterial physiology [14].

It is well known that during pregnancy, a relative state of immunosuppression occurs, which increases the susceptibility of patients to the development of urinary tract infections (UTIs), along with urinary stasis and exacerbated vesico-ureteral reflux. UTIs in pregnancy are primarily caused by Gram-negative bacteria (GNB) [15]. The most commonly implicated pathogens in the literature for UTIs include *Klebsiella spp*, *Proteus spp*, and *Pseudomonas aeruginosa* [16].

Urinary infections caused by multidrug-resistant germs continue to be responsible for significant maternal and fetal morbidity and mortality rates. Thus, the most frequently cited complications associated with these infections are preterm birth, intra-amniotic infections, maternal and neonatal sepsis, or low birthweight [16,17]. Moreover, recurrent or complicated urinary tract infections are associated with significant maternal and neonatal morbidity and mortality [18].

Careful consideration should be addressed to those pregnant patients who previously had recurrent urinary tract infections, complicated urinary tract infections, or who required percutaneous or endoscopic catheterization of the urinary tract during pregnancy, as these risk factors could potentially increase the risk of the development of multidrug resistance and biofilm formation, thus posing a significant challenge for their monitoring during the gestational period.

Literature data on multidrug-resistant urinary tract infections in pregnancy are scarce, and the aim of this retrospective study was to characterize the bacterial spectrum as well as the profile of microbial resistance to antibiotics in cases of urinary tract infections occurring in pregnant women who gave birth in a tertiary hospital. A secondary objective was to assess the impact of multidrug-resistant urinary tract infections on obstetrical and neonatal outcomes.

## 2. Materials and Methods

In this retrospective observational study, we included pregnant patients with or without multidrug-resistant urinary tract infections who gave birth at the “Cuza Vodă” Obstetrics-Gynecology Clinical Hospital in Iasi between January 2019 and December 2023. Screening for asymptomatic bacteriuria in early pregnancy was performed for all patients who were monitored by an obstetrician since the beginning of their pregnancy. We included patients with confirmed MDR UTIs, either symptomatic or asymptomatic. From this study, we excluded underaged patients, cases with unconfirmed urinary tract infection, first and second trimester abortions, cases with incomplete medical records, and patients who did not consent with their data processing at admission.

This study was conducted in compliance with the Declaration of Helsinki, and ethical approval was granted by the institutional review boards of both participating hospitals. Moreover, this study received the ethical approval from the Institutional Ethics Committee of “Cuza Vodă” Obstetrics-Gynecology Clinical Hospital (No. 6778/24.08.2022). All patients provided informed consent before their inclusion in this study, and patient anonymity was preserved throughout the research process.

A total of 371 pregnant patients were included in the analysis and were segregated into the following groups based on the type of bacterial resistance to antibiotics: MDR UTIs (70 patients, group 1), UTIs resistant to one class of antibiotics (108 patients, group 2), UTIs resistant to two classes of antibiotics (102 patients, group 3), and sensitive UTIs (91 patients, group 4).

The following type of data were extracted from electronical medical records: age and demographic characteristics, the presence of immunosuppressive conditions (i.e., the presence of diabetes, HIV/AIDS, immunosuppressive drugs, autoimmune disorders, splenectomy, etc.), personal history of upper or lower urinary tract infections, nephrolithiasis or ureterohydronephrosis, the presence of JJ urinary stents, the presence of a confirmed urinary tract infection, and the antibiotic susceptibility, as well as adverse obstetrical and neonatal outcomes.

The evaluated obstetrical outcomes included preterm birth (delivery of a fetus before 37 completed weeks of gestation), fetal growth restriction or small for gestational age fetuses (inability of the fetus to achieve its genetic potential of growth as defined in the Delphi consensus [19]), intra-amniotic infections (infections occurring within the amniotic fluid, membranes, or placenta during pregnancy), and premature rupture of membranes (before 37 completed weeks of gestation).

The evaluated neonatal outcomes included respiratory distress syndrome (common respiratory system disease in premature newborns previously known as hyaline membrane disease [20]), neonatal intensive care unit (NICU) admission, neonatal infections (any type of newborn infection, i.e., respiratory tract infection, urinary tract infection, etc.), postoperative neonatal death (occurring in the first 24 h after delivery), and late neonatal death (occurring after 24 h after delivery).

We used descriptive statistics for characterizing the microbial spectrum and the clinical characteristics of the patients. Moreover, we compared this type of data between the evaluated groups using chi-square tests for categorical variables and independent *t*-tests for continuous variables.

A multinomial logistic regression model for evaluating the relationship between the type of urinary tract infection and adverse obstetric or neonatal outcomes was used. Results were reported as relative risk ratios (RRRs) and 95% confidence intervals (CIs). These analyses were performed using STATA SE (version 18.5, StataCorp LLC. College Station, TX, USA). A *p*-value of less than 0.05 was considered statistically significant.

## 3. Results

Table 1 comprises a comparative presentation of the UTIs’ microbial spectrum between groups. *E. coli* was the main pathogen implicated in all UTIs from the evaluated groups; however, the percentage was significantly higher for the groups with UTIs resistant to one (67.25%) or two classes of antibiotics (63.51%) compared to the other groups assessed (group 1: 43.58% and group 4: 56.6%, *p* = 0.003). Additionally, the second most frequently identified germ in the urine cultures of these patients was *Enterococcus faecalis*, which also had the highest prevalence in the groups resistant to one (16.59%) and two classes of antibiotics (15.67%). The difference between groups regarding the prevalence of *Enterococcus faecalis* was statistically significant (*p* = 0.04).

*Klebsiella* species were significantly more frequently found in the two aforementioned groups (group 1: 6.60% vs. group 2: 10.19% vs. group 3: 9.62% vs. group 4: 8.58%, *p* = 0.02). It is noteworthy that we encountered two cases of *Pseudomonas aeruginosa* (3.6%) and *Acinetobacter baumanii* (3.6%) only in the group of patients with MDR UTIs.

A total of 13 patients had double JJ stenting, and their UTIs’ microbial spectrum included the following bacteria: *E. coli*—6 patients (46.15%), *Klebsiella* spp.—3 patients (23.07%), *Enterococcus faecalis*—2 patients (15.38%), *Staphylococcus aureus*—1 patient (7.69%), and *Proteus mirabilis*—1 patient (7.69%).

The most frequently encountered resistance was to beta-lactams, fluoroquinolones, and tetracyclines. Among the beta-lactam class, bacteria were often resistant to penicillin, with or without the combination of beta-lactamase inhibitors and to first- to third-generation cephalosporins; however, we encountered only two cases of resistance to carbapenems.

Resistance to ciprofloxacin and norfloxacin (fluoroquinolones) was less frequently found than with agents from the beta-lactamase class, while resistance to tetracyclines and aminoglycosides was moderately present in the resistant bacterial cohort. Additionally, we identified pan-resistance of the pathogens to linezolid (oxazolidinones) and to the combination of trimethoprim–sulfamethoxazole (antimetabolites).

Table 2 comprises a comparative analysis of the clinical and demographic characteristics of the study groups. The examined groups were homogeneous in terms of demographic characteristics, and we did not detect statistically significant differences between them. On the other hand, we found that patients with MDR UTIs had significantly more rates of immunosuppressive conditions (group 1: 34.28% vs. group 2: 10.18% vs. group 3: 7.84% vs. group 4: 5.4%, *p* < 0.001), pyelonephritis, or urinary tract infections during pregnancy (group 1: 15.71% vs. group 2: 3.70% vs. group 3: 3.92% vs. group 4: 0%, *p* < 0.001).

Additionally, these patients exhibited significantly more rates of ureterohydronephrosis (group 1: 22.85% vs. group 2: 5.55% vs. group 3: 6.86% vs. group 4: 4.39%, *p* < 0.001) as well as nephrolithiasis (group 1: 10% vs. group 2: 2.77% vs. group 3: 3.92% vs. group 4: 0%, *p* = 0.03), requiring more procedures such as the placement of JJ stents (group 1: 12.85% vs. group 2: 0.92% vs. group 3: 2.94% vs. group 4: 0%, *p* = 0.01).

Table 3 comprises the results from a multinomial logistic regression that evaluated the association of various types of UTIs with adverse obstetrical and neonatal outcomes. Our regression model results indicated that the presence of MDR UTIs was associated with an increased risk of premature rupture of membranes (RRR: 3.97, 95%CI: 0.40–9.01, *p* = 0.03) and preterm birth (RRR: 2.64, 95%CI: −0.27–8.42, *p* = 0.028), as well as respiratory distress syndrome (RRR: 2.17, 95%CI: 0.14–7.23, *p* < 0.001), and admission to the NICU (RRR: 1.97, 95%CI: 0.32–4.51, *p* < 0.001).

On the other hand, patients with UTIs resistant to two classes of antibiotics showed an increased risk of prematurity, difficult adaptation to neonatal life due to respiratory distress syndrome (RRR: 1.98, 95%CI: 1.38–5.61, *p* = 0.004), as well as admission to the NICU (RRR: 1.47, 95%CI: 0.14–5.08, *p* = 0.04). Only a significantly higher risk of respiratory distress syndrome was encountered in cases of UTIs resistant to one class of antibiotics (RRR: 1.64, 95%CI: 0.22–4.56, *p* = 0.006).

## 4. Discussion

Extended antimicrobial resistance places an additional burden on the healthcare system, and often implies the prescription of more advanced and expensive broad-spectrum antibiotics, which are associated with more side effects and have an uncertain safety profile for mothers and fetuses. The trend of MDR infections in recent years has been upward globally, and the WHO published The WHO AWaRe (Access, Watch, Reserve) antibiotic book in 2022, which includes a series of recommendations regarding antibiotic therapy administration in various clinical scenarios [21]. For urinary infections in pregnancy, treatment recommendations include the use of a narrower-spectrum antibiotic based on urine culture and antibiogram results or rapid improvement of the clinical condition if culture results are not available, as well as a duration of antibiotic treatment of 7 days [21].

In this study, we analyzed data regarding the bacterial spectrum and microbial resistance to antibiotics in cases of urinary tract infections occurring in pregnant women who gave birth in a tertiary hospital in a four-year timeframe. Our results indicated that, in the case of MDR UTIs, the bacterial spectrum mainly included *E. coli*, *Enterococcus faecalis*, and *Klebsiella* species. It is noteworthy that we encountered two cases of *Pseudomonas aeruginosa* and *Acinetobacter baumanii* in this group of patients.

Also, in our cohort of patients, we found almost universal resistance to ampicillin, and this antibiotic is regularly used for the prophylaxis of infections arising from surgical procedures in our institution. On the other hand, we did not identify any cases of XDR or PDR germs in the urine cultures.

These results are in accordance with previously published studies. For example, a cross-sectional study by Asmat et al. evaluated the prevalence of UTIs in 80 pregnant women and characterized their uropathogenic bacterial strains [22]. Their results indicated that *Escherichia*, *Klebsiella*, *Pseudomonas*, *Streptococcus*, *Enterococcus*, and *Staphylococcus* genera were the most frequently encountered in urine cultures, as identified using biochemical characterization. The three strains exhibiting the most significant levels of multidrug resistance were *Pseudomonas aeruginosa* strain UA17, *Escherichia coli* strain UA32, and *Klebsiella pneumoniae* strain UA47 [22].

Another cross-sectional study analyzed urine cultures of 300 pregnant patients and determined the bacterial isolates along with their antibiotic resistance profile. The main uropathogenic determinants were *E. coli*, *S. aureus*, coagulase-negative *Staphylococci* (CoNS), and *Proteus* species. The majority of both Gram-negative and Gram-positive bacteria were resistant to ampicillin [23].

A recent systematic review and meta-analysis conducted by Salary et al. indicated an overall prevalence of both symptomatic and asymptomatic UTIs in pregnancy of 23.9% [24]. However, their true prevalence is difficult to estimate. Several risk factors and physiological changes during pregnancy play an important role in the development and spread of urinary infections. The current literature has identified the following as predisposing factors for UTIs: ethnicity, advanced maternal age, multiparity, urinary tract interventions during pregnancy, personal history of UTIs, immunosuppressive conditions (e.g., diabetes mellitus), anemia, smoking, low education level, or socio-economic status [25,26].

This study confirmed that significantly associated factors with MDR infections include a history of immunosuppression, pyelonephritis, UTIs during pregnancy, maternal urinary tract interventions, and the presence of JJ stents. Additionally, we found an increased frequency of infections with *Escherichia coli*, *Klebsiella* spp., and *Enterococcus faecalis* in patients with JJ stents, a finding that is also confirmed by other studies in the field [16].

Yuan et al. investigated the incidence and microbiological profile of MDR/XDR Gram-negative UTIs, as well as the risk factors for these types of infections, in 1569 kidney transplant patients [27]. The authors showed that 88 patients developed MDR/XDR Gram-negative UTIs, with *Escherichia coli* being the most prevalent uropathogen (62.5%). Almost all MDR/XDR Gram-negative bacteria have shown resistance to first- and second-generation cephalosporins, in addition to monocyclic beta-lactams. The primary risk factor for the development of MDR/XDR Gram-negative UTIs was nosocomial infection. The authors identified that non-fermenting bacterial infections, polycystic kidney disease, and serum creatinine levels exceeding 1.5 mg/dL were significantly distinct between XDR and MDR infections [27].

Another study investigated the association between antibiotic resistance and recurrent urinary tract infection with *Escherichia coli* [28]. Out of 8553 UTIs included in the analysis, 963 were recurrent UTIs. A total of 46.5% MDR UTIs were related to recurrent UTIs, as well as 24.3% XDR UTIs, and 42.5% ESBLs [28].

A prospective observational study examined urinary tract infections associated with catheters in the upper urinary tract among 209 patients, comprising 99 with double-J stents, 81 with nephrostomy, and 29 with internal/external nephroureteral stents [29]. *Escherichia coli* and *Enterococcus* were the predominant bacteria in double-J carriers. MDR microorganisms were isolated in 28.6%, 47.1%, and 58.3% of patients with double-J, nephrostomy, and internal-external nephroureteral stents, respectively. The existence of any form of upper urinary tract catheters and immunosuppression were significant risk factors for the development of MDR UTIs [29].

A secondary objective was to assess the impact of multidrug-resistant urinary tract infections on obstetrical and neonatal outcomes. Maternal complications associated with MDR UTIs include chorioamnionitis, premature rupture of membranes, preterm labor, and anemia [30,31,32].

Our data confirmed an increase in rates of preterm birth and premature rupture of membranes in patients with MDR infections but could not confirm the association between this type of infection and intra-amniotic infections. The risk of preterm birth in pregnant patients who had a urinary tract infection was assessed in a cohort of more than 3 million patients. The authors demonstrated that patients with a UTI during pregnancy were at increased risk of any category of preterm birth (adjusted risk ratios: 1.1–1.4), and that the increased risk was maintained even after patients received antibiotic treatment (aRR: 1.4 for the treated, aRR: 1.5 for the untreated) [33]. A cross-sectional study investigated clinical risk factors associated with premature rupture of membranes in a cohort of 334 patients from Uganda and demonstrated that the significant independent predictor associated with reduced odds of this outcome occurrence was no history of UTIs in the previous month [34].

Neonatal complications associated with UTIs (with or without MDR) include sepsis and pneumonia, intrauterine growth restriction, intrauterine fetal death, and a higher rate of admissions to the neonatal intensive care unit (NICU) [35,36,37].

Our results showed an increased risk of neonatal respiratory distress syndrome due to UTIs caused by MDR bacteria or resistant to two classes of antibiotics, as well as a higher rate of admissions to the NICU for the newborns.

The resistance of bacteria to various classes of antibiotics remains a significant public health issue, and new health policies highlight the need for judicious administration of these medications in various clinical scenarios.

Recent literature has underscored the increased potential of treatments based on bacteriophages, which can induce bacterial lysis and degrade the urothelial biofilm, particularly in cases of MDR UTIs (colistin, vancomycin, etc.) associated with urinary catheters or in forms of recurrent UTIs with complications [38]. For instance, Bhargava et al. assessed the effectiveness of bacteriophage therapy for urinary tract infections in rats, revealing that administering two doses of a phage cocktail at varying concentrations led to the resolution of the infection [39]. Maszewska et al. evaluated the antibiofilm efficacy of phages and formulated a phage cocktail to address the biofilm formation of Proteus mirabilis strains [40]. A three-phage cocktail effectively inhibited biofilm formation and eliminated biofilms of an equal number of strains or 2–3 additional strains when compared to just one phage. The elements of the three-phage cocktail did not inhibit one another’s function.

In the evaluated cohort, we did not detect the presence of bacteria resistant to rescue antibiotics such as vancomycin or colistin; however, we observed a higher rate of resistance to beta-lactams, fluoroquinolones, and aminoglycosides, which justifies a reassessment of local antibiotic prophylaxis protocols for procedures performed during pregnancy or at childbirth.

The results from the following study should be evaluated considering several limitations. The relatively small sample size and the limited number of risk factors considered may restrict the generalizability of the findings. Moreover, the low incidence of MDR UTIs and the absence of PDR or XDR UTIs can constitute a bias of selection that reduces the overall accuracy of the evaluated models. These results are based on data gathered from a single tertiary center. A multicenter cohort study could better outline the MDR profile of UTIs in pregnant patients.

A thorough evaluation of maternal risk factors for UTIs is necessary, as is the use of appropriate antibiotic treatment regimens to prevent the emergence of MDR uropathogens. The increased incidence of pathogens resistant to commonly used antibiotic classes in pregnancy suggests the need for the development of local and national protocols that adapt therapeutic and prophylactic regimens to clinical realities.

## 5. Conclusions

In our cohort of patients, we found almost universal resistance to ampicillin, and this antibiotic is regularly used for the prophylaxis of infections arising from surgical procedures in our institution. On the other hand, we did not identify any cases of XDR or PDR germs in the urine cultures.

Our data confirmed an increase in rates of preterm birth and premature rupture of membranes in patients with MDR infections but could not confirm the association between this type of infection and intra-amniotic infections.

Our results showed an increased risk of neonatal respiratory distress syndrome due to UTIs caused by MDR bacteria or resistant to two classes of antibiotics, as well as a higher rate of admissions to the NICU for the newborns.

## Figures and Tables

**Table 1:** Comparative presentation of the UTIs’ microbial spectrum between groups.

Type of Bacteria	MDR UTIs (70 Patients, Group 1)	UTIs Resistant to One Class of AB (108 Patients, Group 2)	UTIs Resistant to Two Classes of AB (102 Patients, Group 3)	Sensitive UTIs (91 Patients, Group 4)	*p* Value
*Escherichia coli*	37 (43.58%)	63 (67.25%)	60 (63.51%)	71 (56.6%)	0.003
*Enterococcus faecalis*	11 (6.60%)	12 (16.59%)	24 (15.67%)	10 (13.98%)	0.04
*Klebsiella* spp.	11 (6.60%)	14 (10.19%)	6 (9.62%)	4 (8.58%)	0.02
*Staphylococcus* spp.	3 (2.45%)	4 (3.78%)	3 (3.57%)	3 (3.19%)	0.96
*Enterobacter*	1 (1.89%)	5 (2.91%)	3 (3.57%)	1 (2.45%)	0.41
*Streptococcus* spp.	1 (1.89%)	3 (2.32%)	3 (3.57%)	1 (2.45%)	0.75
*Pseudomonas aeruginosa*	2 (3.6%)	0 (0%)	0 (0%)	0 (0%)	NA
*Acinetobacter baumanii*	2 (3.6%)	0 (0%)	0 (0%)	0 (0%)	NA

Legend: MDR—multidrug-resistant; UTI—urinary tract infection; NA—not applicable.

**Table 2:** Comparative analysis of the clinical and demographic characteristics of the study groups.

Characteristic	MDR UTIs (70 Patients, Group 1)	UTIs Resistant to one Class of AB (108 Patients, Group 2)	UTIs Resistant to Two Classes of AB (102 Patients, Group 3)	Sensitive UTIs (91 Patients, Group 4)	*p* Value
Age, years (mean ± SD)	28.12 ± 6.32	29.09 ± 6.60	27.08 ± 6.34	28.26 ± 6.29	0.18
Environment (n/%)	Rural = 32 (51.6%) Urban = 45 (41.8%)	Rural = 66 (62.9%) Urban = 36 (39.1%)	Rural = 42 (43.8%) Urban = 29 (27.2%)	Rural = 58 (56.1%) Urban = 33 (34.9%)	0.76
Parity (n/%)	2.06 ± 2.08	1.56 ± 0.5	1.36 ± 0.48	1.27 ± 0.62	0.44
Immunosuppressive conditions (n/%)	Yes—24 (34.28%)	Yes—11 (10.18%)	Yes—8 (7.84%)	Yes—5 (5.4%)	<0.001
UHN (n/%)	Yes—16 (22.85%)	Yes—6 (5.55%)	Yes—7 (6.86%)	Yes—4 (4.39%)	<0.001
Nephrolithiasis (n/%)	Yes—7 (10%)	Yes—3 (2.77%)	Yes—4 (3.92%)	Yes—0 (0%)	0.03
History of pyelonephritis or lower UTIs (n/%)	Yes—11 (15.71%)	Yes—4 (3.70%)	Yes—4 (3.92%)	Yes—0 (0%)	<0.001
JJ stent (n/%)	Yes—9 (12.85%)	Yes—1 (0.92%)	Yes—3 (2.94%)	Yes—0 (0%)	0.01

Legend: MDR—multidrug-resistant; UTI—urinary tract infection; SD—standard deviation; UHN—ureterohydronephrosis.

**Table 3:** Multinomial logistic regression that evaluated the association between types of UTIs and the occurrence of adverse obstetrical and neonatal outcomes.

Variables		MDR UTIs (Group 1)	*p* Value	UTIs Resistant to one Class of AB (Group 2)	*p* Value	UTIs Resistant to Two Classes of AB (Group 3)	*p* Value
		Risk Ratio, 95%CI		Risk Ratio, 95%CI		Risk Ratio, 95%CI	
Adverse obstetric outcomes	Preterm birth	2.64 (−0.27–8.42)	0.028	0.26 (−0.22–0.74)	0.28	1.78 (0.14–6.88)	0.042
	FGR/SGA	0.33 (−0.23–0.90)	0.25	−0.44 (−1.05–0.96)	0.15	0.23 (−0.20–0.87)	0.22
	Intra-amniotic infections	0.35 (0.07–4.04)	0.14	-	-	-	-
	Premature rupture of membranes	3.97 (0.40–9.01)	0.03	0.13 (−2.07–1.42)	0.90	0.56 (−1.33–1.78)	0.93
Adverse neonatal outcomes	Cesarean birth	0.82 (−1.35–3.01)	0.63	0.21 (−1.44–0.98)	0.76	0.56 (−1.71–1.23)	0.44
	Respiratory distress	2.17 (0.14–7.23)	<0.001	1.64 (0.22–4.56)	0.006	1.98 (1.38–5.61)	0.004
	NICU admission	1.97 (0.32–4.51)	<0.001	1.02 (−0.04–3.23)	0.05	1.47 (0.14–5.08)	0.04
	Neonatal infections	0.18 (−2.75–1.22)	0.86	-	-	-	-
	Postoperative neonatal death	0.15 (−2.86–1.44)	0.76	-	-	-	-
	Late neonatal death	-	-	-	-	0.09 (−2.99–0.86)	0.56

Legend: MDR—multidrug-resistant; UTI—urinary tract infection; CI—confidence interval; FGR/SGA—fetal growth restriction/small for gestational age; NICU—neonatal intensive care unit.

## Data Availability

The datasets are available from the corresponding authors upon a reasonable request.
